# QoS Management and Flexible Traffic Detection Architecture for 5G Mobile Networks

**DOI:** 10.3390/s19061335

**Published:** 2019-03-17

**Authors:** Fernando López Rodríguez, Ugo Silva Dias, Divanilson R. Campelo, Robson de Oliveira Albuquerque, Se-Jung Lim, Luis Javier García Villalba

**Affiliations:** 1 Department of Electrical Engineering, University of Brasília, 70910-900 Brasília, Brazil; fernando.lopez.rodriguez1@gmail.com (F.L.R.); udias@unb.br (U.S.D.); robson@redes.unb.br (R.d.O.A.); 2 Centro de Informática, Universidade Federal de Pernambuco, 50740-560 Recife, Brazil; dcampelo@cin.ufpe.br; 3 School of Software, Hallym University, Hallymdaehak-gil, Chuncheon-si, Gangwon-do 24252, Korea; limsejung@hallym.ac.kr; 4 Group of Analysis, Security and Systems (GASS), Department of Software Engineering and Artificial Intelligence (DISIA), Faculty of Computer Science and Engineering, Office 431, Universidad Complutense de Madrid (UCM), Calle Profesor José García Santesmases, 9, Ciudad Universitaria, 28040 Madrid, Spain

**Keywords:** 5G architecture, multiprotocol label switching, network function virtualization, QoS management, QoS traffic detection, software-defined networks

## Abstract

The next generation of 5G networks is being developed to provide services with the highest Quality of Service (QoS) attributes, such as ultra-low latency, ultra-reliable communication, high data rates, and high user mobility experience. To this end, several new settings must be implemented in the mobile network architecture such as the incorporation of Network Function Virtualization (NFV) and Software-Defined Networking (SDN), along with the shift of processes to the edge of the network. This work proposes an architecture combining the NFV and SDN concepts to provide the logic for Quality of Service (QoS) traffic detection and the logic for QoS management in next-generation mobile networks. It can be applied to the mobile backhaul and the mobile core network to work with both 5G mobile access networks or current 4G access networks, keeping backward compatibility with current mobile devices. In order to manage traffic without QoS and with QoS requirements, this work incorporates Multiprotocol Label Switching (MPLS) in the mobile data plane. A new flexible and programmable method to detect traffic with QoS requirements is also proposed, along with an Evolved Packet System (EPS)-bearer/QoS-flow creation with QoS considering all elements in the path. These goals are achieved by using proactive and reactive path setup methods to route the traffic immediately and simultaneously process it in the search for QoS requirements. Finally, a prototype is presented to prove the benefits and the viability of the proposed concepts.

## 1. Introduction

In the last few decades, the growth in the number of mobile devices has increased mobile network traffic. The diversity of mobile applications such as Voice over IP (VoIP), web navigation, virtual reality, mobile TV, online games, etc., has also expanded the variety of QoS traffic requirements, which drives operators to improve their services. The fifth-generation (5G) of mobile networks is emerging with the purpose of satisfying those increasing demands by providing the highest QoS traffic characteristics such as ultra-low latency, ultra-reliable communication, high data rates, and high user mobility experience [1].

The existing 4G cellular technology developed by 3GPP (Third Generation Partnership Project) [2] has significantly improved the characteristics of the previous cellular technology. However, 4G technology still has a number of limitations. The mobile logical entities of a 4G architecture are based on customized hardware configured and deployed in a static and cost-ineffective manner. The customization limits the elasticity of on-demand provisioning processes and the network innovation cycle. The control and data plane are coupled in the SGW—Serving Gateway—and in the PGW—Packet Data Network (PDN) Gateway, which increases complexity in network management possibilities and limits the network scalability. Additionally, the 4G mobile network data plane is centralized, so the traffic to and from the User Equipment (UE) always has to be routed through the PGW, which can be an inefficient routing alternative when the UEs involved in the communication are close to each other.

To mitigate the weaknesses of the 4G architecture and to achieve the goals for the 5G cellular technology, the works in [3,4,5] have proposed the use of Network Function Virtualization (NFV) in the mobile network architecture. NFV [6,7] technology allows the virtualization of the main entities of mobile architecture, which can be implemented as a software layer placed in less expensive commodity hardware or run in a cloud computing environment. NFV offers a flexible scaling alternative, which can be adapted in order to attend to variations of different demands and provide scaling in a distributed way, while increasing the robustness of the mobile network architecture.

Another widely-proposed alternative to overcome the limitations of the 4G technology is the incorporation of SDN concepts [8,9]. SDN architecture moves the control plane of mobile entities to central devices called controllers, which have a global view of the network and are responsible for managing the data plane of mobile networks. SDN offers a standard open interface to the communication between the controller and the data plane equipment, increasing the network flexibility and programmability and simplifying the data plane elements [10]. However, some disadvantages of a logically-centralized network control are the excessive dependence of every node on the controller, the large amount of information the controller must process, and the additional delay in the specific QoS flow creation, since the packet must wait for the controller response to be forwarded [11].

With SDN and QoS in mind, this paper proposes a new way to address the QoS traffic detection and the QoS traffic management, two of the principal requirements of next-generation 5G networks [12]. If the traffic with QoS requirements is faster and efficiently detected, it can be processed faster. Then, with the specialized QoS treatments, an optimal end-to-end QoS flow with the appropriate QoS characteristics can be created. The results show that the proposed logic is flexible and programmable, and it is applied very closely to the generated traffic (close to the UE), which increases the traffic engineering possibilities.

The architecture proposed by our approach provides a network control application able to route traffic immediately with or without QoS requirements, eliminating the waiting times in the creation of new flows by the controller. The traffic is routed and inspected simultaneously to detect the QoS requirements, providing the necessary information to create a specific QoS path end-to-end. In addition, the architecture reduces the overload on the controller by decreasing the number of messages that must to be processed.

As a proof of concept, a prototype with the proposed logic using Open vSwitches, Mininet, and Ryu Controller has been built. The quantitative result shows an SDN architecture that reduces problems related to the controller high response times, and it is able to route QoS traffic on demand immediately. Additionally, two levels for QoS traffic detection are implemented, one on the edge of mobile network at evolved NodeB (eNB) or next generation NodeB (gNB) and another in centralized specialized elements. The comparative analysis shows a flexible and scalable traffic detection logic, which distributes traffic processing at the borders of the mobile network and, at the same time, reduces the centralized processing requirement, increasing the solution’s robustness. As the architecture is created using OpenFlow, it can take advantage of the traffic engineering possibilities that the SDN implementation provides.

The structure of this paper is as follows. Section 2 presents the considerations of the proposed architecture and the related works. Section 3 provides a complete description of the proposed architecture, starting with the initial configuration, and focused on the 4G eNB or 5G gNB logic proposal, traffic detection tables, and specific Evolved Packet System (EPS)-bearer/QoS-flow creations. In Section 4, the prototype is described and operation examples are provided. Section 5 provides quantitative and qualitative analysis comparing the characteristics of the proposed architecture with current Evolved Packet Core (EPC) and other proposals. Finally, Section 6 presents the main conclusions of this work.

## 2. General Architecture Considerations and Related Works

The next generation of mobile networks will bring deep modifications to the mobile architecture, with the requirement of maintaining interoperability with the previous generations of mobile technology. 5G networks are evolving to incorporate NFV, to move the control to the edge of the mobile network, and to be SDN-capable, increasing the separation between the control and data planes [13].

In [14], an interesting overview of the development of software-defined mobile networks was provided, and the contributions were divided into three main issues: radio access network, with the main objective to coordinate and control the radio resource; mobile control, focused on orchestrating the packet processing, mobility, billing, and service provisioning; and Internet or traffic routing, for which the main target is effective and efficient packet forwarding with the appropriate QoS characteristics. The end-to-end mobile network solution must combine all these objectives to achieve the required 5G performance. Due to it being predominantly a survey work, the work in [14] accordingly lacks a specific approach in some important topics, nor does it present tests or possible validations of the presented subjects. In addition, the work in [14] focused on a mobile access network, but it still had a gap in the mobile core network contributions.

**Figure 1:**
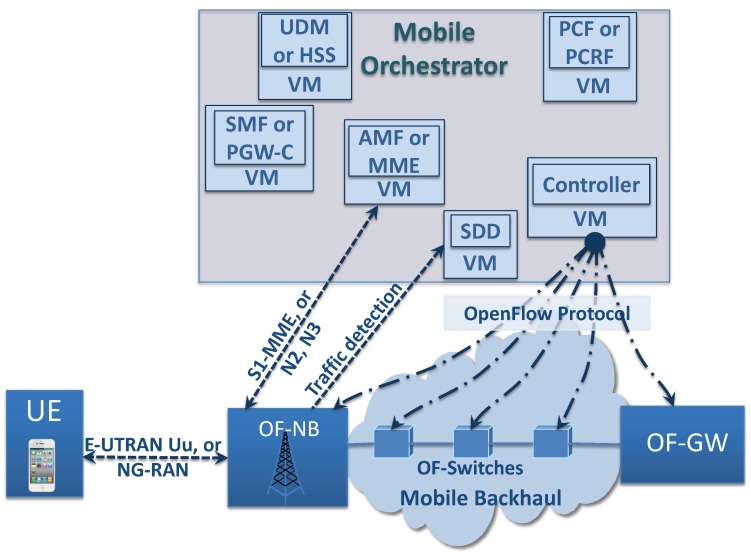
Architectural proposal, visualizing the 4G and 5G mobile control entities. UDM, Unified Data Management; HSS Home Subscriber Server; VM, Virtual Machine; PCF, Policy Control Function; PCRF, Policy and Charging Rules Function; SMF, Service Management Function; PGW-C, PDN Gateway Control; AMF, Access and Mobility Management Function; MME, Mobility Management Entity; SDD, Service Detector Device; OF, OpenFlow; NB, NodeB; E-UTRAN, Evolved UMTS Terrestrial Radio Access Network; UMTS, Universal Mobile Telecommunication System; Uu, UMTS air interface; NG-RAN, Next Generation Radio Access Network; UE, User Equipment.

This paper proposes an architecture that applies SDN to achieve a clear separation between the user and the control planes of the PGW and the SGW mobile elements [15,16,17,18]. The SGW and PGW user plane is implemented by simple OpenFlow [19] switches (called OF-Switch and OF-GW, respectively), and the SGW and PGW control plane is implemented separately in a logical element called PGW-C (PDN Gateway Control). The controller is responsible for managing the data plane of all OF-GW and the physical mobile elements such as the router and switches (implemented as OF-Switches), which are used to transport packets through the mobile core and backhaul (see Figure 1). These separation criteria are applied in 5G [1,20,21], where the user plane of PGW is implemented in a new element called UPF (User Plane Function) and the control plane of PGW is implemented in a new element called SMF (Service Management Function).

As proposed in [22,23,24,25] and in the recently-presented 5G architecture, the NFV concepts are incorporated into the mobile architecture for scalability reasons and demand variation adaption. The election here consists of implementing the control plane of 4G logical mobile elements: PGW-C, MME (Mobility Management Entity), PCRF (Policy and Charging Rules Function), HSS (Home Subscriber Server), and the controller, or even implementing the control plane of 5G logical mobile elements [1,21]: SMF, PCF (Policy Control Function), AMF (Access and Mobility Management Function), and UDM (Unified Data Management) as a Virtual Network Function (VNF) over a cloud environment. On the other hand, the OF-Switch, OF-GW, and base station (4G eNB or 5G gNB) are implemented as physical elements distributed over the mobile domain (see Figure 1).

In [26], a description of how the SDN and NFV concepts can be combined to build a software-defined wireless network was presented. In this work, the implementation of the control plane of the mobile network entities as NFV in a centralized way was proposed, while the data plane was implemented in a distributed way by simple elements managed by a Controller. The described implementation is general and provides only a high-level interaction between the main mobile architecture elements. In particular, it is noted that the QoS requirements are triggered by UE requests, and the mechanisms can be considered fairly centralized. Our proposal uses the SDN and NFV concept in a similar way, but exploring more deeply the way it can be implemented. The detection mechanism presented here is distributed and does not require that the UEs trigger QoS requests. Additionally, the proposed architecture is focused on the reduction of delay in the specific QoS flow creation (on-demand), and changes are not required in the OpenFlow protocol used.

4G eNB or 5G gNB are key elements in the proposed architecture. These elements keep the 4G or 5G mobile access and control interfaces, but incorporate OpenFlow functionality at the same time (these modified elements are called in this work OF-NB). In the proposal, the Controller is responsible for creating all flow entries required to link the traffic between the OF-NB radio interfaces and the backhaul interface. Figure 1 also shows that the OF-NB can optionally implement different types of mobile access interfaces [27,28,29], as 4G E-UTRAN (Evolved UMTS Terrestrial Radio Access Network) or 5G NG-RAN (Next Generation Radio Access Network) interfaces. This work is focused on the backhaul and mobile core network and can be adapted to different access types. As an example of specific QoS flow creation, in Section 3.4, the radio bearer creation is shown using the standard LTE (Long-Term Evolution) procedures with E-UTRAN Uu interface and S1-MEE (Mobility Management Entity) interfaces, as well as the interaction with 4G UE.

In [30], a software-defined architecture for next-generation (5G) wireless systems, namely SoftAir, was introduced. More specifically, the concepts of network function cloudification and network virtualization were exploited, decoupling the radio interface implementation from the base station control, which allows the independent evolution of radio technology. The work in [30] also proposed two levels to detect QoS requirements: (i) the local traffic classifiers placed in a software-based centralized base station at the network edge and (ii) the global traffic learner implemented by the network Controller. Our proposal implements two QoS traffic detection levels as well, but in a significantly different way: (i) traffic detection at OF-NB using standard OpenFlow tables and (ii) specialized software detection devices that can be implemented in a distributed way (not in the Controller), increasing the solution scalability. Furthermore, the data plane proposed here is implemented as completely distributed, and the traffic to be routed does not require being sent to a central device. Finally, the detection process is performed at the same time as the routing process.

A device-to-device communication algorithm able to enhance user quality of experience, by improving the success probability of Internet access in both downlink and uplink directions, was presented in [31]. The Controller is not only able to manage the base station, but also manages the UEs, which can be configured to act as a traffic relay for another UE when the neighboring base stations are congested. Differently, our work is not focused on the mobile access network, but on the mobile backhaul and core network, and the Controller domain is extended to the base stations. The interaction between OF-NB and UEs is performed using 4G standard access interfaces or future 5G access interfaces, and the incorporation of SDN protocols in the UEs is not required, increasing the feasibility of the solution. In particular, a step-by-step procedure is described in order to create an end-to-end path with the QoS requirement.

The proposed mobile architecture uses Multiprotocol Label Switching (MPLS) to implement the data plane. MPLS provides an excellent match between traffic flows and bearers with labels, improving the routing and increasing the traffic engineering possibilities [10]. However, the use of an MPLS data plane is not mandatory, and other alternatives can be used, such as 802.1Q or 802.1ad [32,33] without significant changes in the proposed architecture. It is important to note that the MPLS has 20 bits to identify labels, and 802.1ad only uses 12 bits to identify the VLAN (Virtual Local Area Network); therefore, MPLS increases the number of available identifiers. Additionally, the MPLS protocol is thought to transport traffic through different Layer 2 technologies, which is a useful characteristic in heterogeneous networks.

**Figure 2:**
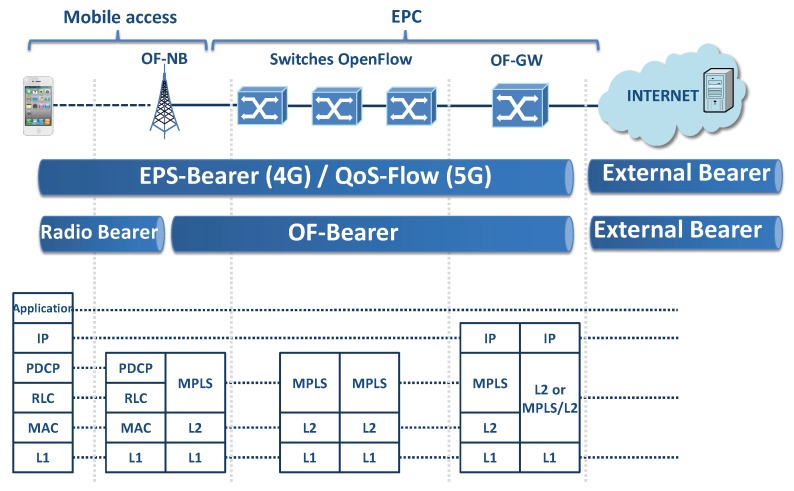
Relationship between Evolved Packet System-Bearer/QoS-Flow with Radio Bearer and the proposed OF-Bearer. MPLS, Multiprotocol Label Switching; EPC, Evolved Packet Core; PDCP, Packet Data Convergence Control; RLC, Radio Link Control.

The use of an SDN data plane generates significant changes, as the use of the GPRS Tunneling Protocol User plane (GTP-U) inside the mobile domain to provide mobility management is no longer necessary [15,32,33,34]. Figure 2 shows the changes in the protocol stack if the proposed SDN-MPLS data plane is combined with the LTE mobile access protocols. As shown, the radio-Bearer between the OF-NB and the UE remains unchanged, but the S1-Bearer and S5/S8-Bearer are unified in a new bearer called the OF-Bearer (managed and created by the Controller). The EPS-Bearer (in EPS terminology) or QoS-Flow(in 5GS terminology [1]) is the concatenation of the Radio Bearer and the OF-Bearer, which is responsible for providing to the UE the appropriate end-to-end QoS communication through the mobile domain. For simplicity, this work references both terms, EPS-Bearer and QoS-Flow, as EPS-Bearer.

**Figure 3:**
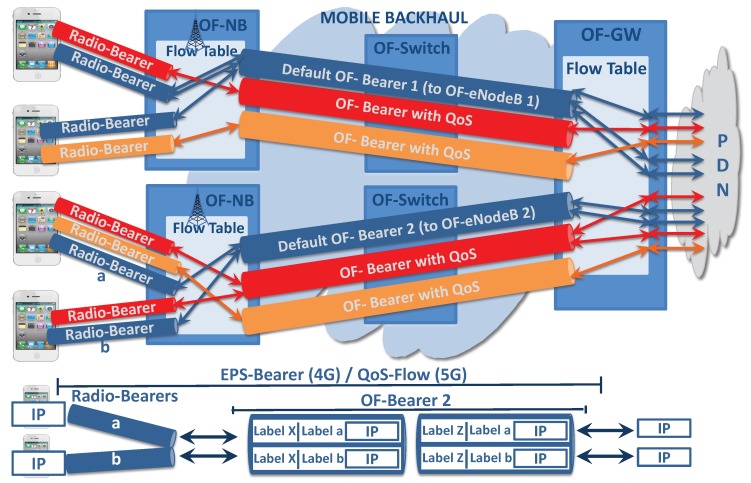
Representation of: MPLS label, EPS-Bearer, Radio Bearer, and OF-Bearer.

Instead of adding a GTP-U header, with the appropriate Tunnel Endpoint Identifier (TEID), to reach the target UE and to identify the appropriate EPS-Bearer/QoS Flow, this work adds two MPLS labels to achieve the same objective. Routing through the EPS-Bearer is achieved by inspecting the two MPLS labels, where the inner MPLS label identifies the UE/Radio Bearer and the outer MPLS label identifies the appropriate OF-Bearer. Figure 3 shows the use of MPLS labels applied to a default EPS-Bearer (Default OF-Bearer 2).

Current mobile networks propose the use of elements called Application Functions (AF) to detect traffic with specific QoS requirements. These elements must be placed in strategic sites, and the traffic to be analyzed must pass through them, thus reducing the solution flexibility; and the AF must be designed to detect the QoS requirement of a specific type of traffic. In the proposed logic, the QoS requirements detection functionality is distributed in the OF-NBs, and it is placed in a flexible and programmable way considering the different user profiles. When this policy detects traffic with QoS requirements, it is addressed to entities (located in central sites such as the data center) that extract the QoS requirements, increasing the traffic detection possibilities and the traffic engineering.

Different from traditional SDN technologies that must wait for the Controller response to route the traffic or use per-configured flow entries to route the traffic, this work proposes a combined logic capable of immediately routing packets using general rules and, at the same time, sending the packets with QoS requirements in order to find the QoS characteristics. As will be described in the next sections, this method increases the routing speed and, at the same time, reduces the impact during the Controller high response times.

## 3. Proposed Architecture

### 3.1. Main Description

Our proposal creates two specialized logics for traffic routing. One is for traffic without QoS requirements, and the other is for traffic with QoS. The first one maintains a central element in the data plane called OF-GW, and all traffic has to be routed through the OF-GW to reach the destination. Key design characteristics to route traffic without the QoS requirement are faster routing, simpler routing policy, and network robustness. On the other hand, traffic with QoS requirements is routed creating specific EPS-Bearers configured to comply with specific QoS traffic requirements such as ultra-low latency, ultra-reliable communication, high data rate, or other QoS requirements.

The proposed architecture uses the two OpenFlow path setup modes [35]: the proactive mode where paths are set up in advance and the reactive mode where the Controller listens to the switches to configure routes on-demand. Both methods have considerations. When the traffic arrives, the proactive mode does not require the Controller actions; it is immediately routed using pre-configured rules, but the packets are sent using general matching criteria. On the other hand, the reactive mode is perfect for creating a specific QoS path on demand, but requires more Controller processing when the packets arrive, and it must wait for the Controller’s response to be routed. Both methods are extensively used in the OpenFlow or SDN academic literature, but this work proposes another option where the traffic with QoS requirements is processed using both operation modes at the same time.

The general idea is summarized as follows. When UE is attached in a OF-NB, it initially has a default EPS-Bearer to OF-GW, which is used to route all traffic without the QoS requirement. When UE sends a packet with the QoS requirement, the OF-NB receives the packet and sends it through two pipelines for simultaneous processing. One pipeline uses a default EPS-Bearer to route the packet immediately (pipeline processing using proactive path setup mode), and the other detects the traffic with QoS requirements and sends a copy of the packet to the new mobile elements called the Service Detector Device (SDD) responsible for extracting the specific QoS requirements. The extracted QoS information is sent to a management entity (as PCRF in 4G or PCF in 5G), and a procedure to create a specific EPS-Bearer with QoS takes place (pipeline processing using reactive path setup mode). Then, the next packets of the same QoS flows are sent using the created specific EPS-Bearer with QoS.

In Section 4.1, an example will be shown in which the prototype initially sends the traffic to the application server through *SW1–SW3–OF-GW 1–SW4– W6*, using the default EPS-Bearer, and when the specific EPS-Bearer with QoS is created, the traffic to the application server is re-routed using the new EPS-Bearer with QoS (sent through the *SW1–SW2–SW5* path).

### 3.2. Initial Configuration

Initially, the mobile network knows the different types of services that the mobile network provides, for example: IMS traffic for VoIP, IMS traffic for video, connectivity with an online game server, mobile TV, etc. (all with specific QoS requirements). Each UE hires a group of these services with specific QoS characteristics to define its user profile. There is a certain number of user profiles, and each UE is associated with one of them (the associations are kept in a database). Initially, the Controller has the mobile network topology, so it is able to configure proactive flow entries in all OF-switches, OF-NBs, and OF-GWs.

The initial proactive steps are the following:

- To create OpenFlow flow entries for all equipment to reach the OF-GW, so they can reach the other OF-GW’s and the mobile service application;
- To route the traffic without QoS requirements, the default OF-Bearer between the OF-NB and the OF-GW is created. All OF-NB must have at least one default OF-Bearer to reach one OF-GW;
- To install the appropriate default route in the OF-GW to reach the PDN network;
- To transport massive services with QoS requirements, the OF-Bearer with QoS between the OF-NB and the OF-GW connected to the services is created;
- To create the traffic detection tables at each OF-NB for all user profiles to detect traffic with QoS requirements (see Section 3.3).

The OF-Bearers can optionally implement queue management to prioritize between different OF-Bearer types, implement bandwidth reservation, guaranteed minimum delay paths, fast recovery mechanisms to carry critical traffic, or other options. All OF-Bearers are univocally identified by an MPLS label, which can change hop-by-hop as in any MPLS network. This MPLS label is the outer MPLS label used to identify UE communication through the specific EPS-Bearer. EPS-Bearers with the same QoS requirement can use the same OF-Bearer (see Figure 3).

### 3.3. OF-NB Logic and Traffic Detection

The proposed logic at the OF-NBs is described for the uplink direction. The proposed traffic detection logic is thought to replace elements of the 3GPP mobile architecture as AF and the Traffic Detection Function (TDF) in a flexible and programmable way and to move the traffic analysis and the billing counters closer to UEs. Moving the control functionalities closer to UE is one of the requirement to achieve the 5G objectives [36,37].

All OF-NB have preconfigured all traffic detection tables, and during the attachment process, each UE is linked with one of the traffic detection tables corresponding to its user profile. The user profile can be obtained during the attachment procedure by querying an appropriate database, or using a non-standard QoS Class Identifier (QCI) value from the HSS, or querying a new 5G database.

**Figure 4:**
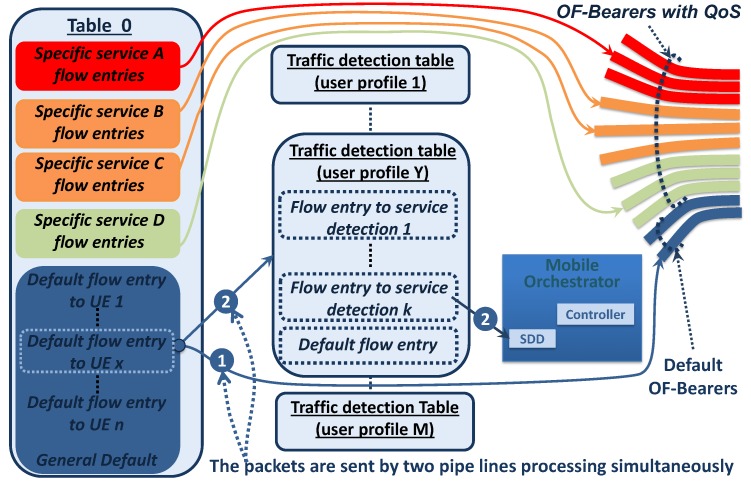
Traffic detection logic at OF-NB.

Figure 4 shows the different OpenFlow tables that compose the uplink forwarding logic or OF-NB; it illustrates M traffic detection tables for M different users profiles, and only the traffic detection table corresponding to user profile Y is detailed. As is depicted in Figure 4, the UE x is associated with the user profile Y, which is linked using the default flow entry to UE x.

In Figure 4, the proposed Table 0, implemented in OF-NB, consists of three types of flow entries:

- Specific service flow entries: This is used to forward traffic with specific QoS requirements from a UE through a specific OF-Bearer with QoS created on demand.
- Default flow entries to UE: Each attached UE has its own default flow entry, and it is used as the default route for all traffic originated in the UE. This flow entry is created during the attachment procedure, and it is responsible for linking the default radio bearer with the default OF-Bearer (creating the default EPS-Bearer for the attached UE). Additionally, this flow entry is responsible for linking the UE traffic with the traffic detection table corresponding to its user profile.
- General default flow entry: This is responsible for dropping the traffic from an invalid source mobile IP address.

Prior to the UE x attachment, the OF-NB has neither a specific service flow entry, nor a default flow entry to UE x. During the specific UE x attachment procedure, the default flow entry to UE x is created. The matching file used by the default flow entries to UE x is based on the source IPv4, the source IPv6 prefix, or the default Radio Bearer, which univocally identifies the UE originator. The default flow entry to UE x links the default radio Bearer with the default OF-Bearer to create the default EPS-Bearer for the UE x. If traffic from UE x does not match with specific service flow entries, it is routed by the default flow entry to UE.

**Figure 5:**
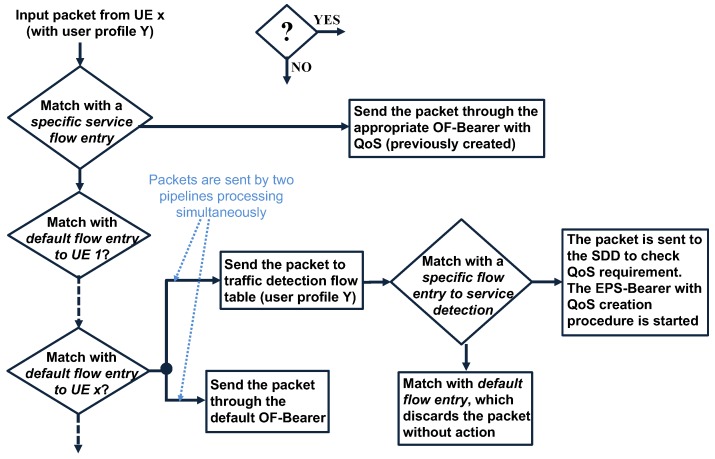
Flow diagram of traffic detection logic at OF-NB.

The proposed logic at OF-NB is detailed in Figure 4, and its flow diagram is described in Figure 5. When the uplink traffic matches with a default flow entry to UE x, the traffic is sent by two simultaneous pipeline processes, one proactively (indicated as one) to route a packet copy immediately as traffic without QoS requirements (using the default OF-Bearer), and the other (indicated as 2) sends the packets to traffic detection table to user profile Y. Next, the implementation of both pipeline processes is explained.

1. The default flow entry to UE x has an instruction of apply-action and, within it, two Push-MPLS actions responsible for univocally identifying the default EPS-Bearer used to send the traffic. The inner MPLS label identifies the UE/default Radio Bearer, and the outer label identifies the default OF-Bearer. Additionally, the instruction of apply-action type has the output-action to the appropriate output port, which sends a copy of the packet to the default OF-Bearer (standard behavior of the apply-action instruction as is specified in [19]).
2. Simultaneously, the default flow entry to UE x has goto tableinstructions, which sends the packet to the traffic detection table for user profile Y. This traffic detection table consists of a group of flow entries responsible for detecting traffic, which requires a specific QoS treatment in accordance with its user profile. When traffic matches with a flow entry to service detection, the appropriate MPLS labels are added, and the packets are sent to the SDD. On the other hand, if there are no matches with a specific detection flow entry, the traffic is dropped without action because the packet was already routed as traffic without the QoS requirement, as was indicated in Step 1.

Continuing Pipeline Processing 2, if the packet is sent to SDD, it processes the packet in search of service characteristics such as the multimedia type, source IP address, destination IP address, protocols, ports used, encoder type, QoS requirement, etc. Next, the SDD sends the service information to the PCRF or PCF mobile element, and the procedure to create the specific EPS-Bearer with QoS requirement is executed. It is important to note that the OF-Bearer with QoS and the Radio Bearer with QoS are linked using the specific service flow entry creating the EPS-Bearer with QoS, as is indicated in Figure 4.

### 3.4. EPS-Bearer with QoS Requirement Creation

**Figure 6:**
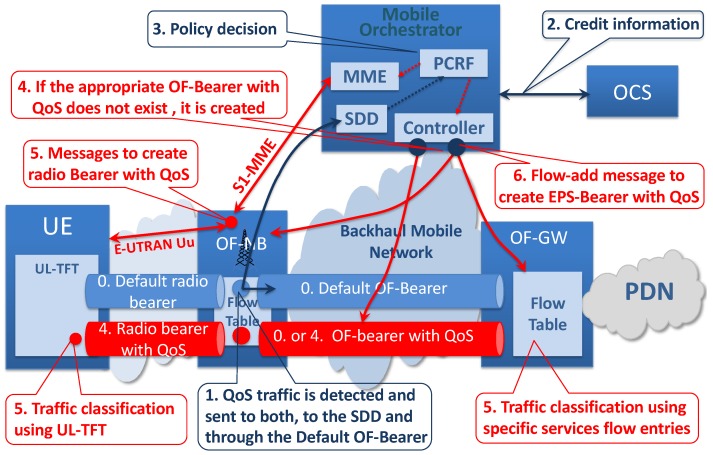
EPS-Bearer with QoS creation. UL-TFT, Uplink Traffic Flow Template; OCS, Online Charging System.

Figure 6 describes the procedure to create a specific EPS-Bearer with QoS considering a 4G UE (a similar procedure can be defined using 5G access technology). As shown, it is not necessary to make changes in the 4G EU. The proposed EPS-Bearer with the QoS creation procedure requires significant changes if it is compared with those defined in the actual PCC (Policy Control and Charging) architecture [38], and it is described next.

1. Initially, the traffic with QoS requirements is immediately forwarded through the default OF-Bearer, and it is sent to the appropriate traffic detection table, which sends the packet to the SDD. The SDD processes the packet and determines the service characteristics. This information is sent to the PCRF (or a new element with its functions), which is responsible for defining the policy to be applied.
2. If additional information is required, the PCRF could make an additional query to other mobile elements. For example, it is possible to make a query to the OCS (Online Charging System) related to the available credit for the service to be transported.
3. With the signaling information, the user profile, and the available credit, the PCRF defines the policy to be applied (the EPS-Bearer characteristics are defined). The policy is communicated to the MME and to the Controller.
4. If there is an OF-Bearer with the appropriate QoS for this traffic, it will be used. On the other hand, if it does not exist, the Controller creates a new OF-Bearer with an appropriate QoS. To do so, the Controller determines the outer labels and creates the appropriate flow entry in each OpenFlow switch involved in the optimal OF-Bearer with the QoS path.
5. Next, performing the following steps, the Radio Bearer with appropriate QoS characteristics is created:
  (a) The MME sends a standard 3GPP bearer setup requestmessage (through the standard S1-MME interface) to the OF-NB indicating the need to create the appropriate radio bearer.
  (b) OF-NB sends the corresponding standard RRC (Radio Resource Control) connection reconfigurationto the UE (standard procedure using the E-UTRAN-Uu interface) indicating that the Radio Bearer with QoS must be created. This message has the required information associated with the QoS parameters and the TFT (Traffic Flow Template) filters to be applied at the UE. These filters incorporate the rules to classify the uplink traffic and forward it to the new Radio Bearer with QoS.
  (c) The UE responds to the OF-NB with a standard RRC connection reconfiguration completemessage.
  (d) The OF-NB responds to the MME with a standard bearer setup responsemessage, completing the Radio Bearer creation stage. At the end of this step, the traffic continues being routed using the default OF-Bearer.
6. The Radio Bearer with QoS and the OF-Bearer with QoS are linked to create the EPS-Bearer with QoS as described next.
  (a) The Controller defines the necessary flow entry in the downlink direction (configured in OF-NB) to link the traffic coming from the OF-Bearer with QoS with the newly-created Radio Bearer with QoS. This step defines the inner MPLS label, which identifies the UE/Radio Bearer.
  (b) The Controller defines the flow entry in the downlink direction that must be applied at the OF-GW to route the QoS service traffic through the OF-Bearer with QoS to the UE. These flow entries are similar to a specific service flow entry put in the OF-NB (see Figure 4), and the matching criteria are based on the service characteristics and target UE IP address (the appropriate two MPLS labels must be added). In this step, the downlink traffic can already be forwarded through the new EPS-Bearer with QoS.
  (c) If not previously configured, the Controller creates the specific flow entries (at OF-GW) in the uplink direction.
  (d) Finally, as can be seen in Figure 4 and Figure 6, the Controller creates the specific service flow entry at the OF-NB to link the traffic from the Radio Bearer with QoS with the OF-Bearer with QoS. This flow entry has its matching criteria based on the service characteristics and the source UE IP address. In this step, the uplink and downlink traffic can already be forwarded, and the EPS-Bearer with QoS is established in both directions.

The specific service flow entries have an appropriate OpenFlow priority, which determines the order in which they are processed. In addition, the specific service flow entries always have higher priority than the default flow entries to UE. Then, if the specific service flow entry exists, the traffic is processed using it (see Figure 6).

It is important to note that the OF-GW are standard OpenFlow switches put at the edge of a mobile network or connected to the application servers. The position where they are placed makes them appropriate to be one end of the default OF-Bearer and, in several cases, one end of the OF-Bearer with QoS, but this is not mandatory. The OF-Bearer with QoS can be created between any pair of OpenFlow switches, for example directly between two OF-NB, as is shown in the prototype (see Figure 8).

### 3.5. Other Considerations Regarding QoS Traffic and Processing

As traffic can be routed directly between two OF-NB, the traffic counter and time counter must be moved to the edge of the mobile access to the OF-NB. In particular, the counters present in the specific service flow entries and in the default flow entries to the specific UE can be used. If during an EPS-Bearer with QoS creation, the OCS informs that the EPS-Bearer to be created has X minutes of credit, the specific service flow entry must be established with an idle-timeout of X minutes. On the other hand, if the credit is related to an amount of remaining traffic, the specific service flow entry should be checked periodically to know when the credit limit is reached.

During normal operation, the traffic without QoS requirements is routed using the default path previously preconfigured, and the Controller interacts only if the traffic has QoS requirements (or in the attachment process). This method reduces the number of OpenFlow messages that the Controller must process, decreasing the Controller overload.

Additionally, since the proposed architecture always routes the packets using the default OF-Bearer immediately, this architecture has less impact during the Controller high response times since traffic continues to be routed through the default OF-Bearer. This behavior improves the robustness of the solution if it is compared with traditional SDN implementations, in which the packets of new flows must wait for the Controller response to be routed.

It is important to note that the proposed traffic detection logic is composed of two main steps, one implemented in a distributed way at OF-NB using the OpenFlow traffic detection table and the second one at SDD using dedicated software to customize the QoS characteristics to be detected. The first step is limited by OpenFlow matching criteria, and the second one is unlimited. Then, the proposed combined detection logic can be considered unlimited. As a practical example, the necessity to identify specific content in the application layer when the packets are sent to a specific server can be considered. In this case, the traffic detection table at OF-NB is responsible for matching when a packet is sent to the server (and to a specific TCP port), as well as for sending the packet to the appropriate SDD. Once the packet is received by the SSD, this element is responsible for extracting the specific application layer requirement.

In order to reduce the processing in the SDD element, this can be implemented in a distributed way in the mobile network domain. In this way, OF-NB can be divided into groups, and depending on their geographical location, they will have a specific SDD associated. Another alternative is to create specific SDDs for the analysis of different types of traffic, and the OF-NB will be responsible for routing the traffic to the appropriate SDD.

Finally, to increment the architecture’s robustness, the Controller function can optionally be divided and put into three different Controllers working together in a complementary way [39]. The optimal Controller separation for the proposed architecture is the use of a Controller for proactive flow configuration, which will be responsible for default OF-Bearer creation, traffic detection tables, and general routing rules. Another Controller for the attachment process will be responsible for the default EPS-Bearer creation and for the association between UE and the traffic detection table. The last one will be responsible for specific OF-Bearer with QoS creation.

## 4. Implementation and Analysis

**Figure 7:**
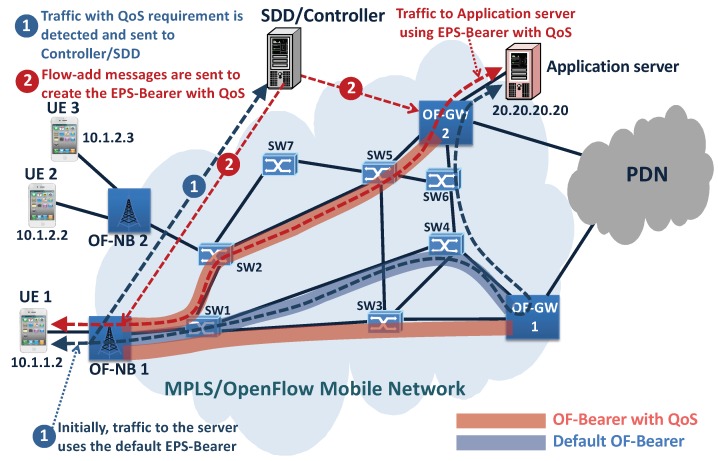
Prototype: Example 1, EPS-Bearer with QoS to the application server. SW, Switch; PDN, Package Data Network.

As proof of concept, a prototype with the topology shown in Figure 7 was created. It is constituted by eleven mobile network elements implemented as OpenFlow switches. Two of the switches represent OF-GW; two represent OF-NB; and seven represent backhaul Switches 1–7 (SW1–SW7). Additionally, four VMs (Virtual Machines) were created: three represent UE, and the fourth represents an application server. The application server represents a service to mobile UEs that requires specific QoS treatments.

Mininet is used for the topology creation; Open vSwitch is used as the software switch that implements the OF-NB, backhaul mobile switches, and OF-GW; while the Controller is implemented using Ryu (all with support for OpenFlow 1.3 or higher due to the use of MPLS).

The prototype implements the traffic detection logic and the EPS-Bearer with QoS creation procedure. Furthermore, it is implemented for both the backhaul and the core mobile network (the mobile access network is outside the scope of this work). Finally, the Controller was implemented as the mobile orchestrator with the functionalities of elements such as PCRF and SDD.

Summarizing, the prototype is built to show the contribution listed next:

- The proposed flexible traffic detection logic operation, giving an example of the different flow entries that compose it.
- The proactive OpenFlow creation, which makes routes for the traffic immediately possible using the default EPS-Bearer without Controller interaction.
- The important characteristic of simultaneous traffic routing and traffic inspecting is detailed. In particular, an example of flow entry is described, which sends the QoS traffic by two pipelines, processing at the same time, one to route the traffic immediately using the default OF-Bearer and the other to process the traffic to identify the QoS requirements.
- It is indicated that the Controller creates an optimal EPS-Bearer with QoS, and the traffic is rerouted using it (examples of specific QoS flow entry to build the EPS-Bearer with QoS are given).
- The proposal uses MPLS, and the GTP-U protocol is not required.
- Finally, as all data plane mobile elements (as OF-GW, OF-NB, and switches) implement OpenFlow, Section 4.2 shows the solution flexibility, creating an optimal path that does not require using an OF-GW.

As described in previous sections, the Controller initially creates the general flow entry to all OF-GW that can reach the other mobile elements, the application servers, and the PDN connection. Next, the Controller creates the default OF-Bearer that connects each OF-NB with the appropriate OF-GW and creates the OF-Bearers with QoS for massive services between OF-NBs and OF-GWs (using the appropriate MPLS labels). Finally, the traffic detection tables are inserted at all OF-NBs.

The following examples start with the UEs attached and with the default EPS-Bearer created. In the case of UE 1, both are created, the default flow entry to UE 1 at eNodeB 1 (the uplink direction) and the default flow entry to UE 1 at OF-GW 1 (the downlink direction). These flow entries are responsible for linking the default Radio Bearer to UE 1 with the default OF-Bearer, building the default EPS-Bearer to UE 1 (a similar configuration is done to UE 2 at eNodeB 2 and OF-GW 2). Examples of the Flow entries are described in the following section.

### 4.1. Example 1, EPS-Bearer with QoS to the Application Server

Figure 7 shows the default OF-Bearer between the OF-NB 1 and the OF-GW 1 (using SW1 and SW4) and the OF-Bearers with QoS between OF-NB 1 and both OF-GWs (proactively created for massive QoS services). The OF-Bearers with QoS between OF-NB 1 and OF-GW 2 represent an optimal path with minimal delay for the OF-NB 1 to reach the application server (Example 1).

Initially, the traffic from the UE 1 to the application server matches the default flow entry to UE 1. Next is shown an example of this flow entry:

*cookie=0x0, duration=35.568s, table=0, n_packets=0, n_bytes=0, ip,**nw_src=10.1.1.2***
*actions=**push_mpls:0x8847,set_field:1010102− >mpls_label,***
***push_mpls:0x8847,set_field:100001− >mpls_label**, dec_mpls_ttl,output:1,**goto_table:15***

It can be seen that the flow entry processes the packets simultaneously through two pipelines: one inserting the two MPLS labels and sending the packets copy through the output Port 1 using the default OF-Bearer to reach the application server (see Figure 7); the other pipeline uses the goto tableaction, which sends the packet to Table 15 (traffic detection table to service profile to UE 1).

When the packet arrives at the traffic detection table, it matches the flow entry to service detection, which detects traffic with the destination to the 20.20.20.20 IP address:

*cookie=0x0, duration=42.918s, table=15, n_packets=0, n_bytes=0, **priority=3500**,ip,nw_**dst=20.20.20.20actions=CONTROLLER**:65535*

As SSD is implemented in the Controller, the flow entry to service detection has an application action of the Controller type; then, detected traffic is sent to the Controller.

The Controller processes the packet-in message, then determines that the traffic must be routed using the OF-Bearer with QoS between OF-NB 1 and OF-GW 2. Next, the Controller creates the specific service flow entry at OF-NB 1 and OF-GW2 to build the required EPS-Bearer with QoS (see Figure 7).

Finally, the specific service flow entry at OF-NB 1 routes the traffic from UE 1 to the application server using the OF-Bearer with QoS.

*cookie=0x0, duration=85.369s, table=0, n_packets=0, n_bytes=0,**priority=4000**,ip,nw_**src=10.1.1.2,***
*ip,nw_**dst=20.20.20.20**actions=**push_mpls:0x8847,set_field:510102− >mpls_label,***
***push_mpls:0x8847,set_field:50102− >mpls_label****, dec_mpls_ttl,**output:1***

The matching criteria used consider both the source UE 1 IP address (10.1.1.2) and the application server target IP address (20.20.20.20). Furthermore, the default flow entry adds two MPLS labels to define the EPS-Bearer with QoS. The 510102 (inner label) identifies the UE 1/Radio Bearer with QoS, and the 50102 (outer label) identifies the OF-Bearer with QoS. Finally, it is important to note that the specific service flow entry has more priority than other types of flow entries. Then, the traffic from UE 1 to the application service matches this specific flow entry and routes the traffic through the EPS-Bearer with QoS, as can be seen in Figure 7.

### 4.2. Example 2, EPS-Bearer with QoS between UEs

**Figure 8:**
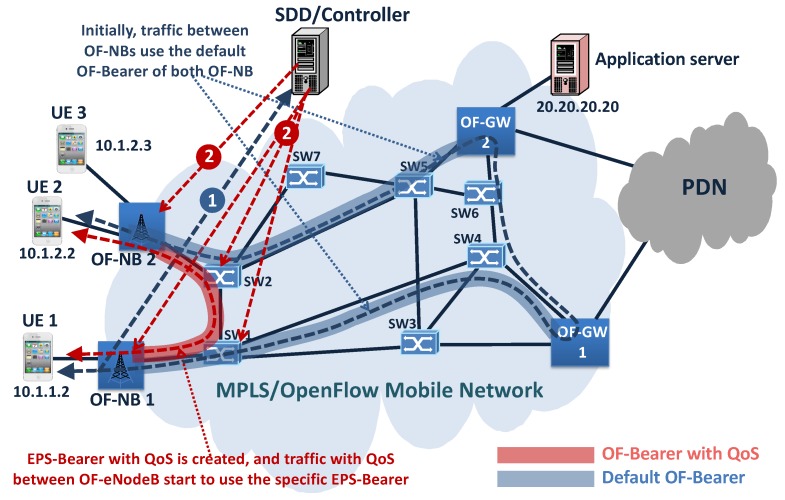
Prototype: Example 2, EPS-Bearer with QoS between UEs.

The second example represents a case when an ultra-low latency communication between UE1 and UE2 is required, creating a specific EPS-Bearer. As can be seen in Figure 8, initially, the traffic between UEs is routed using the UE 1 default OF-Bearer and the UE2 default OF-Bearer. Next, the traffic requirement is detected and sent to the Controller, which decides on the creation of a new specific OF-Bearer with ultra-low latency between OF-NB 1 and OF-NB 2. Then, the Controller inserts the specific service flow entry in OF-NB 1 and OF-NB 2 to link the OF-Bearer created with the Radio Bearer to UE 1 and UE 2, building the EPS-Bearer with ultra-low latency (see Figure 8).

## 5. Quantitative and Qualitative Analysis

In this section, qualitative analyses are carried out to show the proposed architecture characteristics when it is compared with other ones.

### 5.1. QoS Traffic Detection Strategies’ Comparison

Next, a qualitative comparison of different strategies used to detect traffic with QoS requirement in mobile networks is done. The first two use the strategies of the current EPS architecture; the third one is based on NFV, a VNF concept where the logical mobile element is implemented in data centers in a centralized way; and the last one is implemented using the proposed QoS traffic detection logic.

- Mechanism based on the AF detection technique: This refers to the mechanism of the 4G networks that use detection devices called AF, which are put in strategic points of the network. These strategic location points are chosen such that the traffic to be inspected must pass through the AF in its normal way. When the QoS requirements are found, they are sent to the central device (PCRF) responsible for QoS policy implementation. The AF devices are usually placed within or before application servers and are specialized in the detection of different types of traffic. This alternative decrements the flexibility and programmability possibilities due to other types of traffic being able to be routed using different paths. On the other hand, the AF element can be placed in a distributed way, while low addition time is added in the QoS detection process.
- Mechanism based on an element as TDF: This occurs when the detection element is put in the centralized data plane element as PGW in the current EPS architecture. As all traffic goes through the PGW, this method could analyze all types of traffic, but a deep packet inspection could compromise the PGW performance. In this alternative, the large amount of traffic to be processed and the centralized nature of the solution significantly impact the scalability and robustness of the solution.
- Mechanism in centralized data center inspections: Several works proposed [13] the implementation of the data and control plane of mobile elements as VNF over VM using commodity hardware put in data centers. In this alternative, all traffic is sent to a data center, and it is processed to find the QoS requirements. This alternative generates additional delay due to routing of traffic to the data center and requires significant traffic inspection processing. On the other hand, the cloud computing design increments the robustness even if it is implemented in a centralized data center, due to it being an elastic environment able to adapt to the traffic requirement variations.
- Proposed detection mechanism: This alternative is able to send the QoS packet immediately as traffic without QoS requirement, and at the same time, it sends a QoS packet copy to the centralized SDD element to detect the QoS requirement (waiting time is not added). This option increments the robustness due to the QoS traffic continuing to be routed even without the Controller response or without SDD detection (as traffic without QoS requirement). The scalability in this solution is incremented due to the traffic detection functionality is distributed in all OF-NB and all SDD implemented (if only an SDD is implemented can it be considered a Centralized architecture as well, but this could not be an optimal decision). Additionally, this alternative increments the QoS detection possibilities, due to it combining the OF-NB detection and the SDD devices, which are programmable elements able to detect all types of traffic. Finally, this solution could be considered more generic than the previous one. The traffic detection table associated with UE can be changed in the OF-NB at any time, and the new associated detection table can be created to route all the traffic to the SDD element or to a data center (as in the previous alternative).

A summarized qualitative traffic detection comparison can be found in Table 1.

### 5.2. Architectural Comparison

Table 2 shows a qualitative comparison among mobile network architectures implemented using current 4G EPC, using standard centralized SDN OpenFlow where the traffic must wait for the Controller response to be routed, and using the 5G proposed architecture in terms of: (1) forwarding response time; (2) traffic engineering possibilities; (3) GTP-U use requirement; (4) traffic detection flexibility; (5) specific treatment for QoS traffic; and (6) controller processing requirement.

### 5.3. High Controller Response Time and Network Overload Problem Simulation

In order to obtain quantitative results related to the proposed two-pipeline processing, the standard OpenFlow control is implemented over the prototype as well, and the behavior on routing traffic with the specific QoS requirement is compared for both the proposed architecture and standard OpenFlow Control. It is important to note that the standard OpenFlow implemented uses the reactive path setup mode for initial configuration and for traffic without QoS requirement and the reactive setup mode for traffic with QoS requirement; then, when a specific QoS packet arrives, a packet-in message is generated, and the node must wait for the controller response to route the QoS packet.

With the proposed architecture and the standard OpenFlow architecture implemented, some problems related to the communication with the controller are emulated, and the behavior of the architectures when a new packet with specific QoS requirement is sent from OF-NB 1 to the application server is compared. In the comparative analysis, the following problems are considered:

1. Queuing delay in the controller;
2. Controller overload and controller high response time;
3. Requirement of TCP retransmissions in the packet-in request;
4. Delay on the path used for the packet-in request due to link saturation.

It is important to note that the first two mentioned problems affect the packet-in request and the controller response (i.e., a two-way delay), whereas the third and fourth problems could only affect a packet-in request (i.e., a one-way delay).

The tests consider the UE 1 attached and consist of erasing all specific OF-Bearer with QoS (specific service flow entries) and generating traffic with QoS from the OF-NB 1 to the application server with IP 20.20.20.20 (see Figure 7) to measure the average round-trip time between them. This test is repeated for both the proposed and standard OpenFlow controls by adding a variable delay in the communication of OF-NB 1 and OF-GW 1 with the controller. The introduced additional delay ranges from 0 ms–100 ms, with increments of 10 ms. In addition, all tests are done simulating the two types of controller communication delay (the two-way delay and the one-way delay), which are implemented by programming a waiting time in the controller communication.

**Figure 9:**
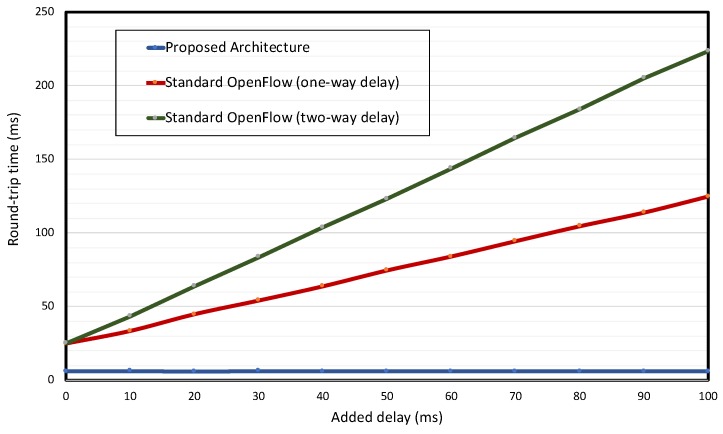
Round-trip times in both architectures and for both additional delay types.

The results are shown in Figure 9. As can be seen, the round-trip time between the hosts is not affected in the proposed architecture. This is due to the fact that the specific QoS packets do not have to wait for the controller response to be routed; they are routed using the default OF-Bearer preconfigured during the attachment process (see Figure 7). On the other hand, if the standard OpenFlow architecture is used, the round-trip time between the hosts is linearly affected, the worst case being when a delay is added in both directions of controller communication (two-way delay).

Another important point is that even without the additional delay, the standard OpenFlow architecture presents a round-trip time 19 ms bigger than that in the proposed architecture. This delay represents two waiting times for the controller responses in the standard OpenFlow architecture, one in the OF-NB 1⟶application server direction and the other in the application server⟶OF-NB 1 direction. The value of 19 ms is lower than that of a real case, because in the prototype, the controller is implemented very close to the nodes. If a real network is considered, the delay curves for the standard OpenFlow architecture significantly move up in the graph due to the propagation delay.

## 6. Conclusions

The next 5G mobile network generation is evolving to incorporate the NFV concept, to move the control to the edge of the mobile network, and to incorporate the SDN concepts, increasing the separation between the control and data plane. This work follows this mobile evolution, particularly working with the weaknesses and strengths of the SDN network and also incrementing the use of this technology in the mobile network architecture. As the programmability is put over all mobile element, more specialized logics can be created.

This work proposes a specialized logic to route the traffic without QoS requirements, which is created using proactive rules. This logic is designed to route the traffic immediately using a routing policy that is as simple as possible. To route traffic without QoS requirements, the data plane of the proposed mobile architecture continues using the centralized element called OF-GW. Then, traffic without QoS must always be routed using an OF-GW. It is important to note that all equipment that is part of control plane is implemented as simple OpenFlow switches, making it easy to replace the switches. The data plane is distributed between more elements, improving the mobile network robustness, and all OpenFlow switches can be configured to act as an OF-GW.

Additionally, a specialized logic to route the traffic with QoS requirements using proactive and reactive methods at the same time is created. The proposed logic is designed to create a specific EPS-Bearer able to use the optimal path with specific QoS characteristics, thereby, satisfying the 5G mobile network objectives. This logic is composed of a programmable and flexible method for detecting traffic with QoS requirements that are applied closer to the UE. The architecture created does not add delays during the EPS-Bearer with QoS creation or the QoS traffic detection. Initially, the packets with QoS requirements are sent by two pipelines processing at the same time, one immediately routing a packet copy through the default OF-Bearer; the other is used to detect the QoS requirements creating the appropriate specific EPS-Bearer with QoS.

The proposed control plane follows the concepts used by the 5GS architecture implementing the mobile control element using the NFV concept. This technology allows the virtualization of the main control entities of mobile architecture, which are implemented as a software appliance placed on less expensive commodity hardware or run in the cloud computing environment. NFV offers a flexible scaling alternative, which can be adapted to the demands’ variations and provide scaling in a distributed way, while increasing the robustness of the mobile network architecture.

Finally, the proposed mobile backhaul and mobile core network architecture are created to interact optionally with a future 5G UE, or 4G UE, utilizing the NG-RAN procedures or the standard procedures using the E-UTRAN-Uu and S1-MME 3GPP interfaces.

## Figures and Tables

**Table 1:** Comparison of QoS traffic detection strategies. AF, Application Function; TDF, Traffic Detection Function.

	AF Mechanism	TDF Mechanism	Centralized Data Center	Proposed Mechanism
Robustness	Low	Low	Good	Very good
Scalability	Good	Low	Very good	Very good
QoS processing	Distributed	Centralized	Centralized	Distributed/Centralized
Traffic detection possibilities	Good	Good	Very good	Very good
Additional delay during QoS detection	Low	Low	Intermediate	Low

**Table 2:** Comparison of mobile network architectures. GTP-U, GPRS Tunneling Protocol User plane.

	EPC Architecture	Standard OpenFlow	Proposed Architecture
Forwarding response time	Immediate	Intermediate	Immediate
Traffic engineering possibilities	Good	Very good	Very good
GTP-U requirement	Required	Required	Not required
Traffic detection flexibility	Good	Good	Very good
Specific treatment for QoS traffic	Intermediate	High	High
Controller processing requirement	N/A	very High	Intermediate
